# Two for one: targeting BCMA and CD19 in B-cell malignancies with off-the-shelf dual-CAR NK-92 cells

**DOI:** 10.1186/s12967-022-03326-6

**Published:** 2022-03-14

**Authors:** Gils Roex, Diana Campillo-Davo, Donovan Flumens, Philip Anthony Gilbert Shaw, Laurens Krekelbergh, Hans De Reu, Zwi N. Berneman, Eva Lion, Sébastien Anguille

**Affiliations:** 1 Laboratory of Experimental Hematology, Vaccine & Infectious Disease Institute (VAXINFECTIO), Faculty of Medicine and Health Sciences, University of Antwerp, 2650 Edegem, Belgium; 2 Division of Hematology, Antwerp University Hospital, Drie Eikenstraat 655, 2650 Edegem, Belgium; 3 Center for Cell Therapy & Regenerative Medicine, Antwerp University Hospital, 2650 Edegem, Belgium

**Keywords:** Bispecific, Dual, Chimeric antigen receptor, NK-92, Off-the-shelf, Leukemia, Lymphoma, Myeloma

## Abstract

**Background:**

Chimeric antigen receptor (CAR) T-cell therapy has proven to be a valuable new treatment option for patients with B-cell malignancies. However, by applying selective pressure, outgrowth of antigen-negative tumor cells can occur, eventually resulting in relapse. Subsequent rescue by administration of CAR-T cells with different antigen-specificity indicates that those tumor cells are still sensitive to CAR-T treatment and points towards a multi-target strategy. Due to their natural tumor sensitivity and highly cytotoxic nature, natural killer (NK) cells are a compelling alternative to T cells, especially considering the availability of an off-the-shelf unlimited supply in the form of the clinically validated NK-92 cell line.

**Methods:**

Given our goal to develop a flexible system whereby the CAR expression repertoire of the effector cells can be rapidly adapted to the changing antigen expression profile of the target cells, electrotransfection with CD19-/BCMA-CAR mRNA was chosen as CAR loading method in this study. We evaluated the functionality of mRNA-engineered dual-CAR NK-92 against tumor B-cell lines and primary patient samples. In order to test the clinical applicability of the proposed cell therapy product, the effect of irradiation on the proliferative rate and functionality of dual-CAR NK-92 cells was investigated.

**Results:**

Co-electroporation of CD19 and BMCA *CAR* mRNA was highly efficient, resulting in 88.1% dual-CAR NK-92 cells. In terms of CD107a degranulation, and secretion of interferon (IFN)-γ and granzyme B, dual-CAR NK-92 significantly outperformed single-CAR NK-92. More importantly, the killing capacity of dual-CAR NK-92 exceeded 60% of single and dual antigen-expressing cell lines, as well as primary tumor cells, in a 4h co-culture assay at low effector to target ratios, matching that of single-CAR counterparts. Furthermore, our results confirm that dual-CAR NK-92 irradiated with 10 Gy cease to proliferate and are gradually cleared while maintaining their killing capacity.

**Conclusions:**

Here, using the clinically validated NK-92 cell line as a therapeutic cell source, we established a readily accessible and flexible platform for the generation of highly functional dual-targeted CAR-NK cells.

**Supplementary Information:**

The online version contains supplementary material available at 10.1186/s12967-022-03326-6.

## Background

Genetic engineering of T cells with a chimeric antigen receptor (CAR) has demonstrated high clinical activity in several B-cell malignancies [1]. This has led to the recent regulatory approval of several CAR-T-cell products, either targeting CD19 or B-cell maturation antigen (BCMA) [2–7]. Due to its ubiquitous expression on malignant B-cells, CD19 was an evident choice as a target antigen for B-cell leukemia and lymphoma. Depending on the cell product, overall response rates and complete responses range from 52 to 85% and 40 to 59%, respectively [1, 7]. These impressive results sparked the development of CARs for a myriad of other malignancies and led to the recent wave of BCMA-targeted CARs under clinical investigation for multiple myeloma (MM). BCMA is highly expressed on myeloma cells, whereas its presence on healthy cells is restricted to lower levels on mature B-cells and plasma cells [8]. Its expression profile, combined with its vital role in the proliferation, survival and drug resistance of MM cells makes BCMA an excellent target for MM.

Despite remarkable initial response rates mediated by CD19- and BCMA CAR-T cells in different hematological malignancies, a majority of treated patients ultimately relapsed [1, 9]. A considerable fraction of these relapse cases can be attributed to the selection and outgrowth of a small tumor cell population with downregulated or absent target antigen expression [10–13]. Subsequent administration of CAR-T cells targeting other cell surface antigens, such as CD20 and CD22 in B-cell leukemia, can reinstate remission [14–16].

Due to the developmental relationship between B-cells and plasma cells, it comes as no surprise that some overlap exists between CD19 and BCMA expression. In this regard, several groups confirmed the presence of BCMA antigen on a subset of CD19^+^ cells from B-cell leukemia and lymphoma patient samples, as recently reviewed by Dogan et al. [17]. Similarly, a small, less differentiated BCMA^+^CD19^+^ MM subpopulation was recently described, which was shown to be chemoresistant and to have cancer stem cell-like properties [18, 19]. Therefore, combinatorial approaches employing chemotherapeutics together with CD19- and BCMA-targeted CAR therapies are already under investigation in the context of MM [8, 20, 21].

Combinatorial multi-target therapies in the clinic are currently relying on simultaneous or sequential administration of the different CAR-T-cell products [8, 22–25]. However, co-administration of two single-CAR-T-cell products could lead to one product outcompeting the other, indicating that a single effector cell carrying multiple CARs would be more desirable [22]. There are, however, several drawbacks related to the production of single-antigen targeted autologous CAR-T cells [26]. First, the quality of autologous, patient-derived T cells is generally poor due to the detrimental effects of prior treatments [26, 27]. Second, CAR-T-cell production is already associated with a substantial price tag [28], further reducing financial accessibility when adding a second production run. Third, current manufacturing processes are lengthy, making them unsuitable for patients with aggressive disease. The use of allogeneic T cells derived from healthy donors could represent a solution but this field is still in its infancy ([29–32] and ClinicalTrials.gov identifier: NCT04142619) and their applicability is beset by the need for additional genetic modifications in order to reduce the risk of graft-versus-host disease (GvHD) [33]. Another population of effector cells that is gaining attention as an alternative for T cells is the natural killer (NK) cell. NK cells are of particular interest due to their innate anti-tumor capacity mediated through their activating receptors, their favorable cytokine profile and the lack of GvHD [33]. However, primary NK cells generally face the same issues for clinical application as T cells, namely their limited ex vivo expansion capacity and population heterogeneity, in addition to their considerable resistance to genetic modification [34]. In contrast, the allogeneic NK-92 cell line provides a continuously expanding, homogeneous and easily engineerable off-the-shelf source of NK cells that is increasingly used in the clinic. This is exemplified by the fact that the NK-92 cell line has obtained FDA investigational new drug application status and by the growing number of clinical trials using (CAR-modified) NK-92 cells [33].

Here, we present a dual-targeting strategy with NK-92 co-expressing two complete CD19- and BCMA-specific CARs. We demonstrate that simultaneous transfection of multiple CAR-encoding mRNAs is feasible and results in high dual-CAR expression. Dual-CAR NK-92 cells efficiently recognize and eliminate single- and double-positive target cells, including primary tumor cells, even at low effector to target ratios. Furthermore, we confirm that dual-CAR NK-92 cells maintain their functionality after gamma-irradiation, which supports their off-the-shelf clinical applicability.

## Methods

### Primary cells, cell lines and culture conditions

Human Burkitt’s lymphoma cell lines Daudi and Namalwa were purchased from the American Type Culture Collection. The NK-92 cell line was purchased from the German Collection of Microorganisms and Cell Cultures. U266 is a multiple myeloma cell line kindly gifted by Dr. Wilfred T.V. Germeraad (GROW School for Oncology & Developmental Biology, Maastricht University, Maastricht, The Netherlands). The enhanced green fluorescent protein (eGFP)-transduced erythroleukemia cell line K562 was generated in-house [35] (parental K562 was a kind gift from Dr. Cedrik Britten [R&D Oncology, GlaxoSmithKline, Stevenage, UK]). CD19.eGFP- and BCMA.eGFP-modified K562 (referred to as CD19-K562 and BCMA-K562, respectively) were kind gifts from Dr. Michael Hudecek (Hudecek Lab, University of Würzburg, Würzburg, Germany). Daudi, Namalwa, U266, K562, CD19-K562 and BCMA-K562 were maintained in Iscove’s Modified Dulbecco’s Medium (IMDM; Life Technologies) supplemented with 10% fetal bovine serum (FBS; Gibco). NK-92 cells were maintained in GlutaMAX alpha Minimum Essential Medium (α-MEM; Gibco) supplemented with 12.5% FBS and 12.5% horse serum (Gibco) (NK-92 medium) and 100 U/mL recombinant human (rh) interleukin (IL)-2 (ImmunoTools). All cell lines were maintained in logarithmic growth phase at 37 °C in a humidified atmosphere supplemented with 5% CO_2_. For potential future clinical applications, NK-92 cells need to be irradiated prior to administration to avoid further cell proliferation in vivo. Therefore, where specified, NK-92 were irradiated with 10 Gy in the X-RAD320 (Accela) 4 h after *CAR* mRNA electroporation, and were incubated another 20 h before use in subsequent in vitro assays. Primary B-cell acute lymphoblastic leukemia (B-ALL) blasts were isolated from the peripheral blood of two patients using CD19^+^ magnetic selection (Stemcell Technologies) and cryopreserved for further use. In contrast, assays against MM were performed on freshly isolated bulk bone marrow mononuclear cells (BMMNC) obtained from bone marrow samples from MM patients.

### Generation of CAR-expressing NK-92

Two second generation CAR constructs against the target antigens CD19 and BCMA were designed using the same backbone: a CD8α leader peptide, an antibody-derived single-chain variable fragment (scFv), a CD8α hinge and transmembrane domain (referred to as “CD8”), a 4-1BB (CD137; referred to as “BB”) co-stimulatory region and CD3ζ signaling domain. The sequence of the fully human scFv against BCMA was obtained from patent WO2016090320A1 (Seq No. 85), whereas the fully human scFv targeting CD19 was found in patent US20100104509A1 (47G-4). The synthetic genes CD8-CD19-CD8BBz and CD8-BCMA-CD8BBz were assembled from synthetic oligonucleotides and/or PCR products. The fragments were inserted into pST1-Rhamm (GeneArt, Thermo Fisher Scientific). Subsequent production of CAR-encoding mRNA through in vitro transcription (IVT) was previously described [36, 37]. Prior to electroporation, 200 µL of 25 × 10^6^ NK-92 cells/mL in Opti-MEM (Life Technologies) was mixed with nuclease-free water (IDT, Leuven, Belgium) (mock NK-92), 50 µg/mL *BCMA CAR* mRNA (BCMA-CAR NK-92), 50 µg/mL *CD19-CAR* mRNA (CD19-CAR NK-92) or both (dual-CAR NK-92) in a 4 mm cuvette (ImmunoSource). Cells were pulsed using a Gene Pulser Xcell (Bio-Rad) with a time constant protocol (300 V, 12 ms) and recovered in NK-92 medium without IL-2 for use in downstream applications. CAR surface expression was evaluated 24 h later by staining 2 × 10^5^ cells with 300 ng rhBCMA-FITC or 1 µg rhCD19-PE (AcroBiosystems) for 1 h at 4 °C prior to acquisition on a CytoFLEX flow cytometer (Beckman Coulter).

### NK-92 degranulation

CD107a was used as a marker of NK-92 degranulation upon target recognition. Cell membranes of target cells were labeled with CellTrace Violet (Molecular Probes, Invitrogen) according to manufacturer’s instructions. Of the stained cells, 2 × 10^5^ were subsequently co-cultured with transfected NK-92 cells at an effector to target ratio of 1:2 in U-bottom 96-well plates for 5 h. At the start of the incubation period, 10 µL anti-CD107a-PE (BD Biosciences) was added to each well. As a protein transport blocker, 1× monensin (Biolegend) was added 1 h into the co-culture. Samples were acquired on the FACSAria II (BD Biosciences) and gates were set based on appropriate fluorescence-minus-one controls.

### Flow cytometric cytotoxicity assays

In case of tumor cell lines and primary B-ALL cells, target cells were membrane labeled with PKH26 (Sigma Aldrich) or CellTrace Violet directly prior to co-culture. Twenty-four hours after electroporation, CAR-transfected NK-92 and membrane labeled target cells were distributed in a U-bottom 96-well plate at different E:T ratios, briefly spun down (120*g*, 2 min) to optimize cell contact and incubated for 4 h. Cell pools were subsequently stained with 7-AAD (BD Biosciences) and annexin V-FITC (Invitrogen) or -APC (BD Biosciences) and measured on the a CytoFLEX or FACSAria II flow cytometer, respectively. The proportion of cytotoxicity was calculated based on the fraction of live cells (double negative for 7-AAD and annexin V) using the formula: % cytotoxicity = 100 – (live target cells with effector cells/live target cells without effector cells)*100. Specific lysis was further calculated by subtraction of cytotoxicity induced by the mock NK-92 control.

Due to the limited quantity of MM cells in patient bone marrow aspirates, we conducted a flow cytometric killing assay based on counting beads using complete BMMNC. CellTrace Violet labeled CAR NK-92 were co-cultured for 4 h with 5 × 10^4^ BMMNC at different E:T ratios. Co-cultures were subsequently harvested and stained with LIVE/DEAD Fixable Near-IR (Life Technologies), anti-CD38-FITC (clone HIT2), anti-CD45-BV650 (clone HI30), anti-CD56-BV785 (clone 5.1H11), anti-CD19-APC (clone HIB19; all Biolegend), anti-CD3-PE-Cy7 (clone UCHT1) and anti-CD138-PE-CF594 (clone MI15; BD Biosciences). Precision counting beads (Biolegend) were added immediately prior to acquisition on a NovoCyte Quanteon (Agilent) to determine absolute counts of viable CD138^+^CD38^+^ MM cells. Cytotoxicity against primary MM cells was calculated using the formula: % cytotoxicity = 100 – (absolute number of viable MM cells in treated wells/mean absolute number of viable MM cells in untreated wells)*100 [38].

### Quantification of granzyme B and IFN-γ secretion

Transfected NK-92 and target cells were resuspended at a concentration of 1 × 10^6^/mL and 100 µL of each was added to a U-bottom 96-well plate in triplicate. After 4 or 16 h of incubation, supernatant was harvested for the quantification of granzyme B or IFN-γ using enzyme-linked immunosorbent assay (ELISA; R&D Systems and Peprotech, respectively) according to manufacturer’s instructions. After development of the plates, absorbance was measured on a VICTOR^3^ multilabel plate reader (PerkinElmer).

### Statistical analysis

Flow cytometric data was analyzed using FlowJo v10.7.1 software (TreeStar Inc). GraphPad Prism 9 (GraphPad Software) was used for graphical presentation and statistical analysis of the data. For normally distributed endpoints, a two-tailed unpaired t test was performed for comparison between two groups. Alternatively, data consisting of three or more groups were analyzed using one-way analysis of variance (ANOVA), performing Dunnett’s or Tukey’s post hoc tests for multiple comparisons where appropriate. Results were considered statistically significant with a *p* value < 0.05. * indicates *p* < 0.05, ** indicates *p* < 0.01, *** indicates *p* < 0.001 and **** indicates *p* < 0.0001.

## Results

### Generation and specificity of BCMA- and CD19-CAR NK-92

**Fig. 1:**
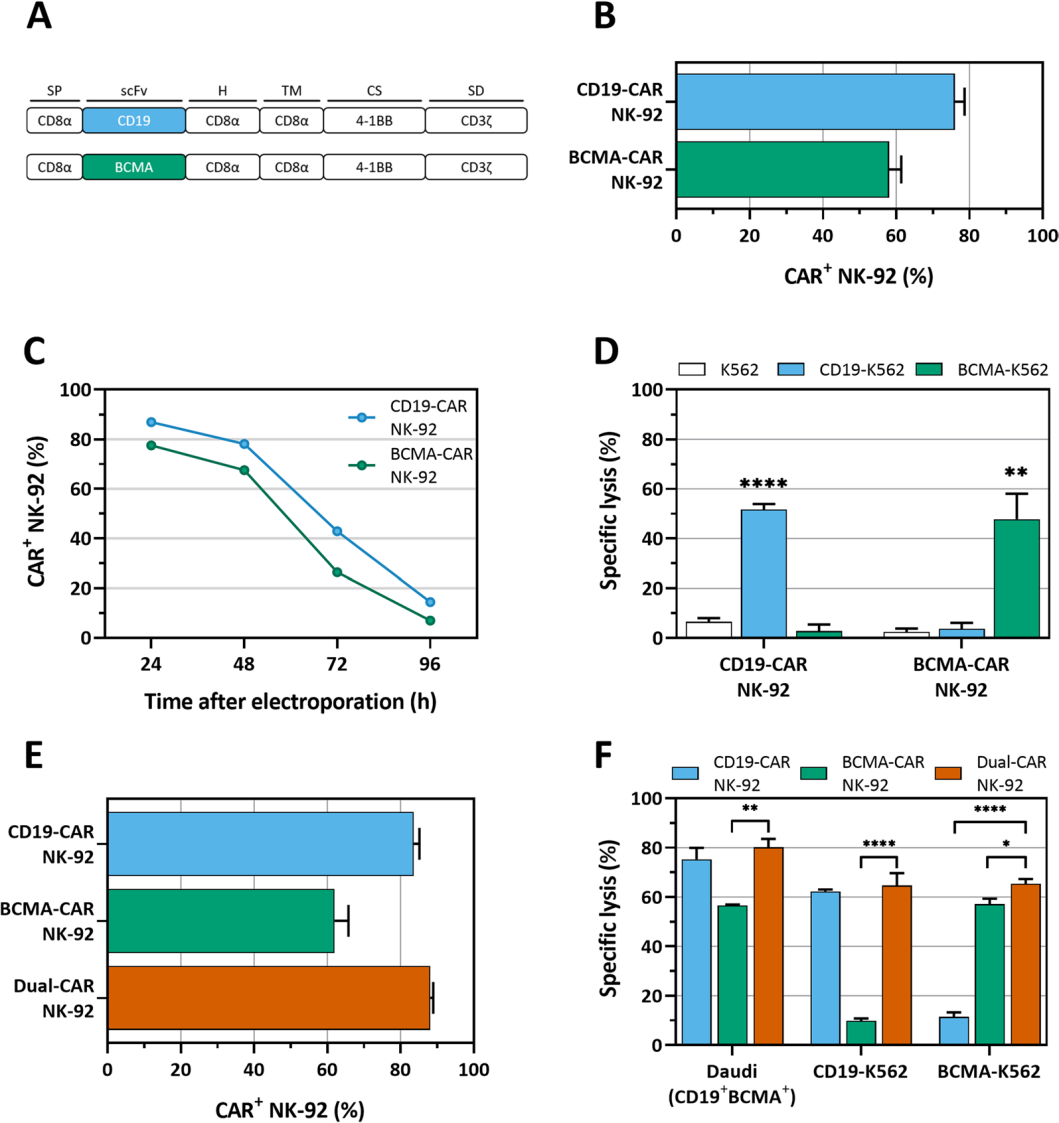
Generation and functional validation of dual-CAR NK-92. **A** Structural composition of the BCMA- and CD19-specific CARs used for the dual-CAR approach. **B** High CAR expression in transfected NK-92 cells, 24 h after electroporation with 50 µg/mL CAR-encoding mRNA (N = 26–28). **C** CD19 and BCMA-CAR surface expression kinetics in NK-92 over four days (N = 1). **D** Only target cells expressing cognate antigen are lysed, confirming CAR specificity. Statistical analysis was performed using ANOVA with Tukey’s correction for multiple comparisons (N = 3). **E** CAR expression of NK-92 transfected with mRNA encoding one (CD19-CAR NK-92 and BCMA-CAR NK-92) or both CARs (dual-CAR NK-92). Expression for dual-CAR NK-92 represents cells positive for both CARs (N = 19). **F** Dual-CAR NK-92 lyse BCMA^+^CD19^+^ (Daudi), as well as single BCMA^+^ (BCMA-K562) or CD19^+^ (CD19-K562) tumor cells (N = 3). Statistical analysis was performed using ANOVA with Dunnett’s correction for multiple comparisons relative to the dual-CAR NK-92 condition. *p < 0.05. **p < 0.01. ****p < 0.0001. BCMA: B-cell maturation antigen; CAR: chimeric antigen receptor; CS: co-stimulatory domain; H: hinge domain; scFv: single-chain variable fragment; SD: signaling domain; SP: signal peptide; TM: transmembrane domain

For this study, we designed two IVT plasmid vectors encoding either a or CD19- or BCMA-specific CAR. The extracellular antigen-recognition domains of our CAR constructs consisted of a fully human scFv. The remainder of the CAR building blocks, i.e. the CD8α hinge and transmembrane domain, and intracellular 4-1BB costimulatory and CD3ζ signaling domains, were identical to those used in the first-in-class CD19 and BCMA CAR-T cell therapies, tisagenlecleucel [39] and idecabtagene vicleucel [40], respectively (Fig. 1A). NK-92 cells were electroporated with either BCMA- or CD19-specific *CAR* mRNA in order to generate BCMA-CAR NK-92 or CD19-CAR NK-92, respectively. We were able to efficiently transfect NK-92 cells, obtaining 76.0 ± 2.7% CD19-CAR^+^ and 58.1 ± 3.3% BCMA-CAR^+^ NK-92 cells 24 h after electroporation (Fig. 1B). CAR expression peaked at 24 h and gradually decreased towards baseline over the course of four days (Fig. 1C). Antigen specificity is an important requirement for CAR products to avoid off-target toxicity. We evaluated specificity of BCMA-CAR NK-92 and CD19-CAR NK-92 through a flow cytometric cytotoxicity assay against parental K562 (control), and K562 overexpressing CD19 (CD19-K562) or BCMA (BCMA-K562). BCMA-CAR NK-92 and CD19-CAR NK-92 showed lysis of K562 cells expressing their cognate antigens (47.8 ± 10.3% and 51.7 ± 2.3%, respectively), but left parental K562 cells and those transduced with irrelevant antigen unharmed, confirming antigen specificity (Fig. 1D). By depriving the transfected NK-92 cells of IL-2 for 24 h, the background toxicity towards the parental NK-sensitive K562 cells was reduced to negligible levels (Fig. 1D), confirming that the observed cytotoxic effects are largely attributable to CAR-driven activation of NK-92 cells.

Following confirmation of the specificity of each CAR, we investigated dual-CAR-expressing NK-92 (dual-CAR NK-92) in the context of antigen escape. NK-92 cells were simultaneously loaded with equal amounts of *BCMA-CAR* and *CD19-CAR* mRNA. Dual-CAR NK-92 cells displayed high CAR expression with 88.1 ± 0.8% of the cells expressing both CARs (Fig. 1E), confirming that electroporation of two CAR constructs does not lead to a competitive reduction in CAR expression (Additional file 1). Importantly, dual-CAR NK-92 are capable of recognizing and eliminating CD19-K562 and BCMA-K562, displaying only one target antigen, with equal efficiency to their single-CAR-expressing counterparts (Fig. 1F), suggesting they are capable of overcoming antigen escape. Moreover, cytotoxicity of dual-CAR NK-92 cells towards the Daudi lymphoma cell line, expressing both CD19 and BCMA, was high (80.1 ± 3.4%) and compared favorably to that of their single antigen-expressing counterparts (CD19-CAR NK-92 and BCMA-CAR NK-92: 75.2 ± 4.7% and 56.5 ± 0.4%, respectively), confirming that dual-CAR loading is not detrimental to the cytotoxic function (Fig. 1F).

### Antigen-specific degranulation and activation of dual-CAR NK-92

**Fig. 2:**
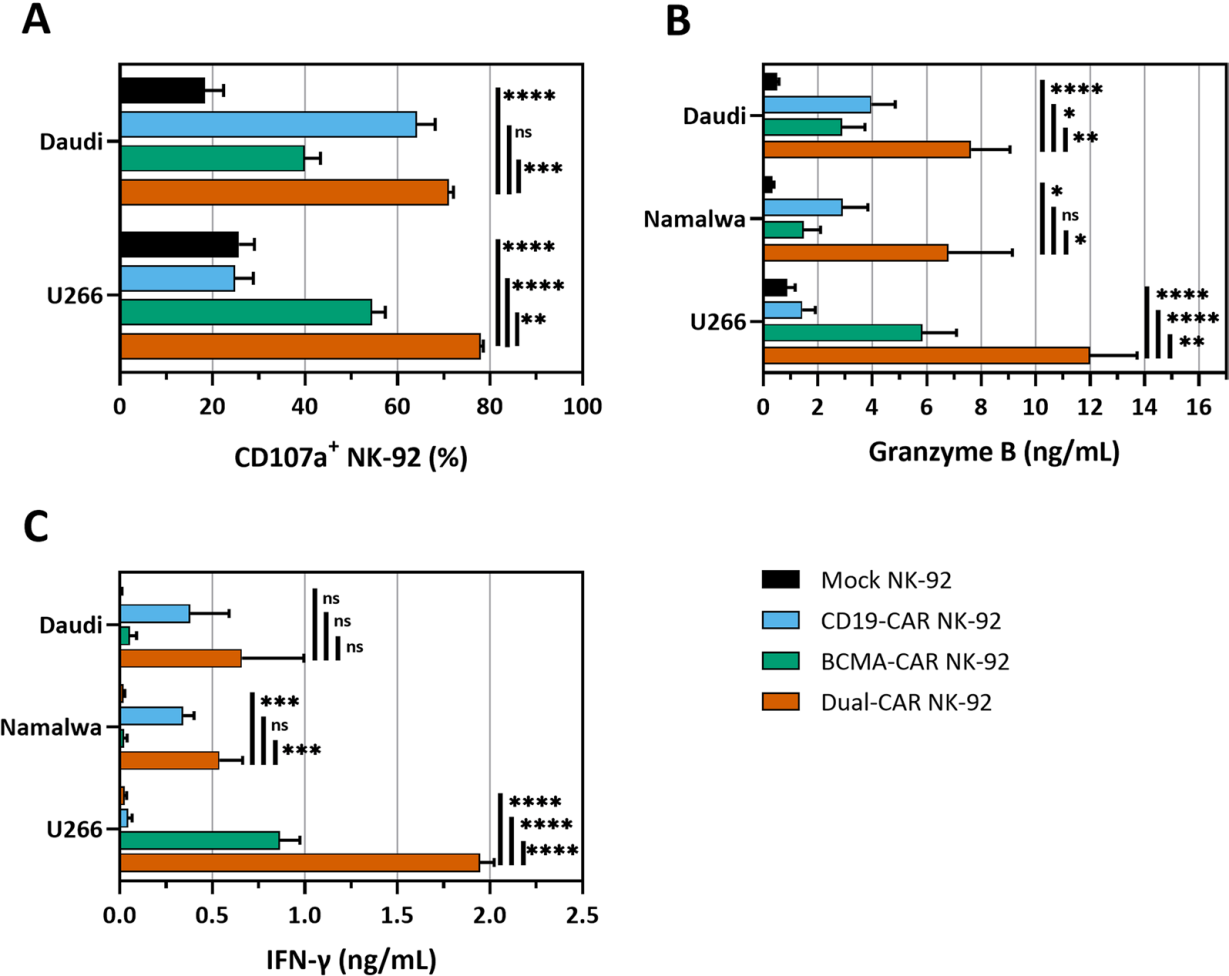
Degranulation and activation of dual-CAR NK-92. **A** CD107a expression demonstrated antigen-specific degranulation of CAR-engineered NK-92 during 5 h of co-culture at a 1:2 E:T ratio (N = 3). **B** High granzyme B secretion by dual-CAR NK-92 in the supernatant of 4 h co-cultures (1:1 E:T ratio; N = 3–6). **C** Dual-CAR NK-92 also significantly secrete IFN-γ upon activation (16 h co-culture at 1:1 E:T ratio; N = 3). Statistical analysis was performed using ANOVA with Dunnett’s correction for multiple comparisons with dual-CAR NK-92 as a reference. ns, not significant; *p < 0.05; **p < 0.01; ***p < 0.001; ****p < 0.0001. BCMA: B-cell maturation antigen; CAR: chimeric antigen receptor

In order to quantitatively assess the activation status of CAR-loaded NK-92 cells, we first evaluated the degree of CD107a surface expression upon NK cell degranulation, a marker for identification of NK activity (Fig. 2A). The intrinsic anti-tumor activity of NK-92 cells observed in the background levels of CD107a surface expression in mock NK-92 cells, which was slightly more pronounced against the NK-sensitive U266 cells compared to the NK-resistant Daudi cells (25.8 ± 3.3% and 18.4 ± 4% CD107a^+^, respectively). Mock NK-92 (25.7 ± 3.3% CD107a^+^) and CD19-CAR NK-92 (24.9 ± 3.9% CD107a^+^) displayed equal levels of CD107a expression in response to CD19^−^ BCMA^+^ U266, indicating that CAR transfection does not impact their natural tumor reactivity. CD107a expression was significantly upregulated in dual-CAR NK-92 cells compared to BCMA-CAR NK-92 cells co-cultured with U266 cells (78.0 ± 0.5% and 54.4 ± 2.9% CD107a^+^, respectively). A similar observation was made between CD19-CAR NK-92 cells and dual-CAR NK-92 cells against Daudi cells (64.2 ± 4.0% and 71.0 ± 1.1% CD107a^+^, respectively).

These findings were further corroborated by the detection of secreted granzyme B in co-culture supernatant in the presence of the relevant antigen, but not in the absence thereof (Fig. 2B). Generally, dual-CAR NK-92 cells significantly secreted more granzyme B against Daudi (7626 ± 1433 pg/mL), Namalwa (6801 ± 2346 pg/mL) and U266 (12,008 ± 1710 pg/mL) compared to CD19-CAR NK-92 cells (3972 ± 873 pg/mL, 2909 ± 926 pg/mL and 1425 ± 478 pg/mL, respectively) and BCMA-CAR NK-92 cells (2894 ± 831 pg/mL, 1482 ± 615 pg/mL and 5842 ± 1251 pg/mL, respectively). Furthermore, IFN-γ secretion by dual-CAR NK-92 cells (Fig. 2C) was also considerably elevated in comparison to CD19-CAR NK-92 cells against Daudi cells (660 ± 334 pg/mL vs 382 ± 209 pg/mL, respectively) and Namalwa (539 ± 126 vs 344 ± 57 pg/mL, respectively), and in comparison to BCMA-CAR NK-92 cells against U266 (1946 ± 77 pg/mL vs 866 ± 107 pg/mL, respectively). Taken together, these results verify that both CARs are capable of robustly activating NK-92 cells, separately as well as combined.

### Irradiation effectively halts NK-92 proliferation, but does not affect cytotoxicity

**Fig. 3:**
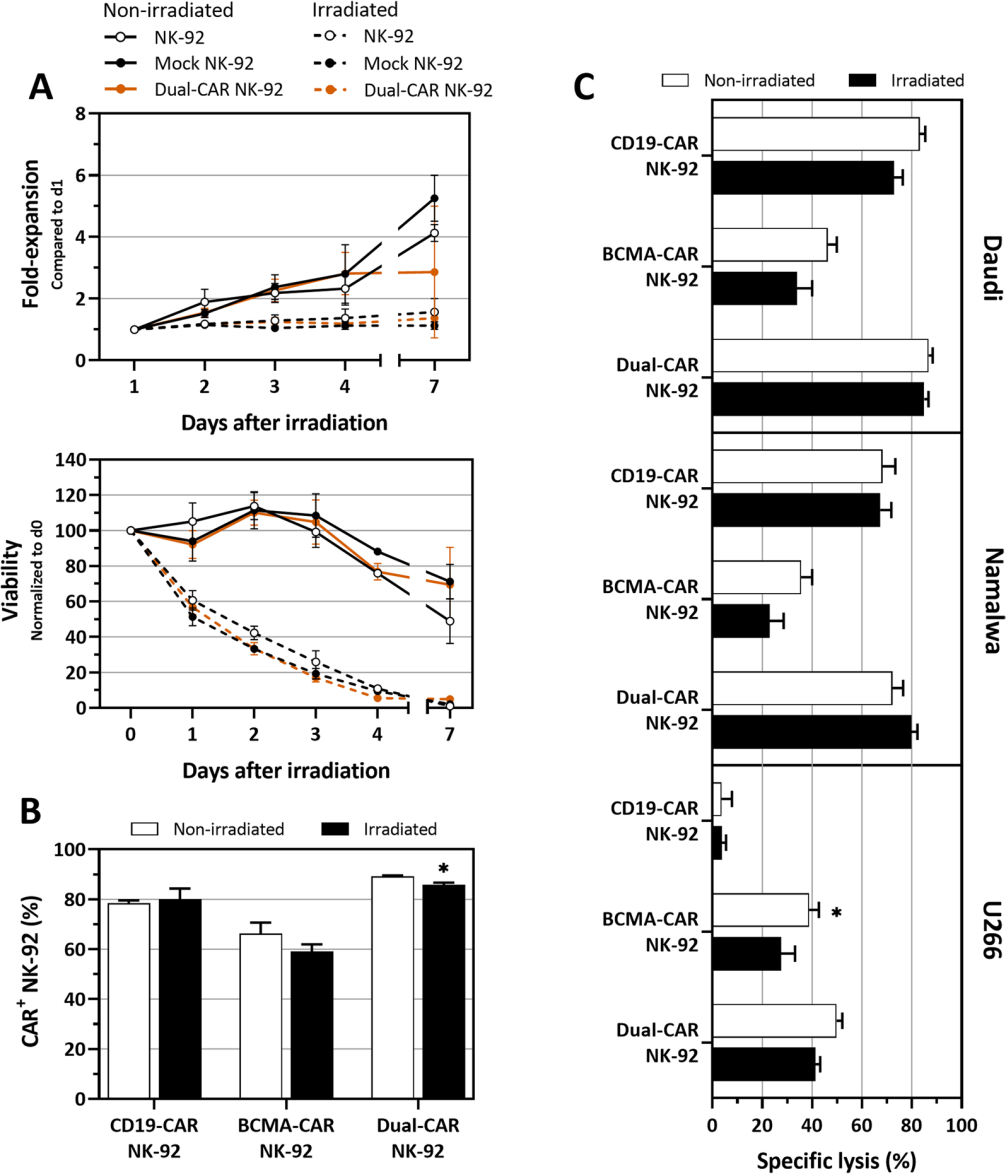
The effect of irradiation on proliferation, viability, CAR expression and functionality of dual-CAR NK-92. **A** Proliferation of NK-92 cells (top) was successfully inhibited after irradiation and viability (bottom) gradually declined over the course of a week (N = 3). Follow-up on day 5 and 6 was not performed and, therefore, not shown in the graph. **B** Peak CAR expression (24 h post electroporation) is maintained following irradiation (N = 3). **C** Despite irradiation, cytotoxic activity of NK-92 towards Daudi, Namalwa and U266 cells was largely preserved (4 h co-culture at 1:1 E:T ratio). Statistical analysis in B and C between non-irradiated and irradiated conditions was performed using an unpaired, two-tailed student t test. *p < 0.05. BCMA: B-cell maturation antigen; CAR: chimeric antigen receptor

The NK-92 cell line requires inactivation prior to administration in order to prevent in vivo proliferation. This is usually achieved by gamma-irradiation, with 10 Gy being established as the recommended dose [41, 42]. Therefore, we irradiated CAR-transfected NK-92 cells 4 h after electroporation with 10 Gy and tracked viability and cell count up to 1 week. As shown in Fig. 3A, non-irradiated parental NK-92, mock NK-92 and dual-CAR NK-92 cells showed similar growth rates, whereas their irradiated counterparts did not proliferate. Additionally, we saw a steady decrease in viability of the irradiated cells over the course of a week, leaving less than 5% of viable cells at the end of day 7, relative to the start of the culture (Fig. 3A). To exclude any potential inhibitory effects on the functional properties of NK-92 cells caused by irradiation [27, 41, 43], we next investigated CAR expression levels and cytotoxic effector function of irradiated single- and dual-CAR NK-92 cells (Fig. 3B and C). As shown in Fig. 3B, 24 h after CAR mRNA electroporation, irradiated NK-92 cells maintained high expression of CD19 and BCMA single-CARs and dual-CAR (80.0 ± 4.3%, 59.1 ± 2.9% and 85.8 ± 0.9%, respectively), similar to non-irradiated CD19-CAR, BCMA-CAR and dual-CAR NK-92 cells (78.5 ± 1.1%, 66.2 ± 4.4% and 89.2 ± 0.4%, respectively). As observed in non-irradiated CAR NK-92, surface expression of the CAR is maximal at 24 h after mRNA electroporation and gradually decreases in the following days due to the transient nature of mRNA (Additional file 2).

Figure 3C depicts the specific lysis of irradiated and non-irradiated CAR NK-92 after a 4 h co-culture with Daudi and Namalwa lymphoma cell lines (both CD19^+^ and BCMA^+^), and with the U266 myeloma cell line (CD19^−^ and BCMA^+^) at an E:T ratio of 1:1. In terms of anti-tumor killing activity of double-positive target cells, no significant differences between non-irradiated and irradiated CAR NK-92 cells were observed (Fig. 3C). Irradiation had no impact on the lytic activity of CD19-CAR NK-92, BCMA-CAR NK-92 and dual-CAR NK-92 against Daudi (72.9 ± 3.4%, 33.9 ± 6.1% and 85.0 ± 1.7%, respectively) or Namalwa cells (67.3 ± 4.4%, 23.0 ± 5.5% and 79.8 ± 2.4%, respectively). Likewise, irradiated dual-CAR NK-92 cells were still capable of efficiently lysing U266 cells (41.4 ± 1.9%) compared to non-irradiated dual-CAR NK-92 cells (49.8 ± 2.3%). Moreover, irradiated dual-CAR NK-92-mediated killing capacity against Daudi (85.0 ± 1.7%) and Namalwa (79.8 ± 2.4%) matched or exceeded that of CD19-CAR NK-92 cells (72.9 ± 3.4% and 67.3 ± 4.4%, respectively). Regarding U266 cells, irradiated dual-CAR NK-92 considerably outperformed the BCMA-CAR NK-92 condition (41.4 ± 1.9% vs. 27.5 ± 5.6%, respectively). Altogether, irradiation effectively inhibits proliferation and persistence of CAR NK-92 while CAR expression and performance are comparable to non-irradiated controls.

### Cytotoxicity of dual-CAR NK-92 towards primary tumor cells

**Fig. 4:**
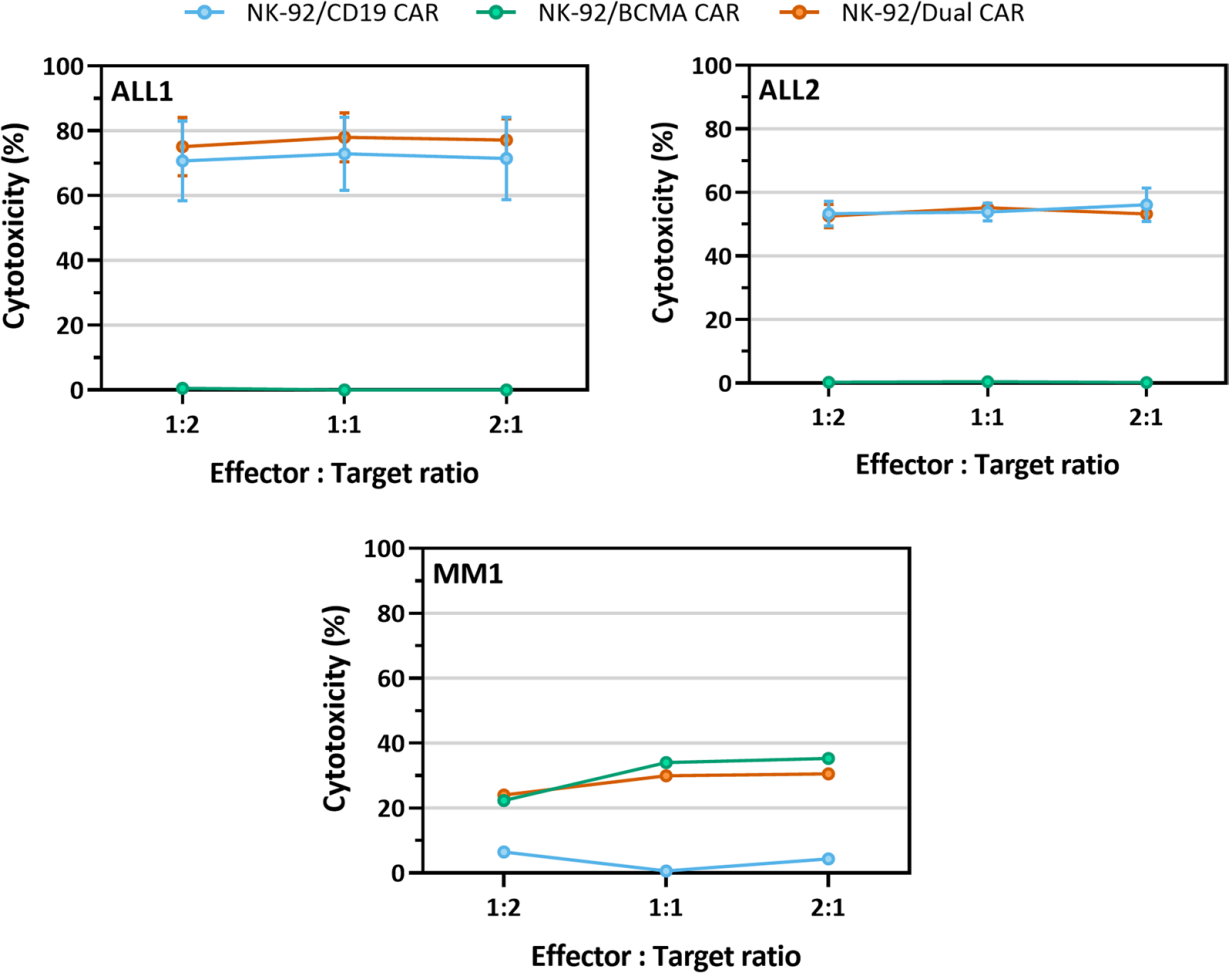
Lysis of primary tumor samples by CAR NK-92. High cytotoxicity of CAR-engineered NK-92 towards two primary B-ALL (N = 2) and one primary MM (N = 1) tumor samples after a 4 h co-culture at different E:T ratios. ALL#, B-ALL sample number. MM#, MM sample number

Finally, we investigated whether our dual-CAR NK-92 cells could also eradicate primary tumor samples (Fig. 4). Comparable to our CD19^+^ tumor cell line models, primary B-ALL blasts were effectively killed by CD19-CAR NK-92 and dual-CAR NK-92 at the different E:T ratios examined (Fig. 4; e.g., ALL1 at a 1:1 E:T ratio, CD19-CAR: 72.9 ± 11.3% and dual-CAR: 78.0 ± 7.6%). Of interest, we could not observe any lysis mediated by BCMA-CAR NK-92 cells, indicating that the activity was CD19-CAR-mediated and not the result of any natural cytotoxic activity of NK-92 cells towards primary ALL cells. Similarly, both BCMA-CAR NK-92 and dual-CAR NK-92 killed primary MM cells at all tested E:T ratios while CD19-CAR NK-92 left them unharmed. Collectively, these results support the finding that simultaneous expression of two CARs does not impede the killing capacity of the NK-92 and that they possess cytotoxic activity not only towards cell lines but also towards primary tumor cells.

## Discussion

CAR-T-cell therapy has demonstrated immense therapeutic potential in hematological malignancies, including B-ALL, B-cell lymphoma and MM, even in patients in whom all standard treatment options have been exhausted. Yet, cancer cells exploit several escape mechanisms to thwart this potent immunotherapy, eventually leading to relapse in a large fraction of patients. Two main types of relapses after CAR-T-cell therapy can be discerned, one being the result of downregulation or complete loss of the target antigen on the tumor cell surface resulting in an inability of the CAR-T cells to recognize the tumor cells (antigen-negative relapses) and the other being the result of the loss of (functional) CAR-T cells; in this case, antigen expression is retained, hence the term antigen-positive relapses. There is now an intensive search for strategies to overcome the problem of antigen-negative as well as antigen-positive relapses. One way to tackle the problem of relapse due to antigen loss is to target more than one antigen [14–16, 44, 45], whereas the second problem could be addressed by producing and re-administering a second batch of CAR-T cells. However, the latter solution is cumbersome given the complex logistics and lengthy duration of autologous CAR-T-cell manufacturing, which is sometimes not possible due to the low numbers or low quality of patient-derived T cells. This problem could in turn be addressed by using an unlimited, off-the-shelf source of functionally active effector cells, such as the NK-92 cell line.

Here, we tackled the aforementioned root causes of relapse following CAR-T-cell therapy by developing a dual-CAR NK-92 cellular therapeutic targeted towards CD19 and BCMA. The reasons to select these two particular targets are obvious; CD19 and BCMA are highly relevant in the context of CAR-T-cell therapy for B-cell hematological malignancies, and, up until now, only CD19- and BCMA-targeted CAR-T-cell products have received regulatory approval. Furthermore, CD19 and BCMA are expressed across different stages in the development of B-cells to mature plasma cells. Hence, dual targeting of CD19 and BCMA offers the prospect of a broadly applicable therapeutic product that can be used in a spectrum of B-cell hematological malignancies ranging from B-cell leukemia and lymphoma to multiple myeloma. Some antigens are co-expressed on cells at the same developmental stage. This is, for example, the case for CD19 and CD20 on B cells; dual-CD19/CD20 CAR-T cells are now under active investigation in B-cell leukemia and lymphoma [46–49]. Similarly, for MM, combinatorial approaches with BCMA and other plasma cell surface antigens such as CD38 are under development [45, 50–54]. In vivo models investigating these dual CAR strategies consistently showed superior tumor clearance and prevention of antigen escape, offering the prospect for deeper and more durable clinical responses [44, 48, 53, 55].

Very recently, Luanpitpong et al. described a dual-CAR NK-92 approach similar to ours, but using a different combination of antigens (CD19 and CD138) and using lentiviral transduction as CAR loading strategy [56]. Here, the two fully human CAR constructs were introduced in the NK-92 by means of mRNA electroporation. In contrast to lentiviral transduction, mRNA electroporation is a rapid, simple, relatively low-cost and highly efficient method for gene transfer in human cells. Boissel et al. previously applied the mRNA electroporation technology for introduction of a single CD19 CAR in NK-92 cells, reaching transfection efficiencies of approximately 50% [57, 58]. CD19 *CAR* mRNA electrotransfection of primary human NK cells yielded comparable results [59, 60]. In this study, we confirmed that mRNA electroporation is a suitable method for CAR loading of NK-92 cells, with either the CD19-CAR and BCMA-CAR being expressed at high levels. In addition, for the first time, we demonstrated that this technology can be used in the NK-92 therapeutic cell source to simultaneously introduce two different CAR constructs without hampering the expression of either CAR molecule. Despite its obvious advantages, such as potentially reducing the duration of severe side effects in treated patients, the temporary CAR expression following mRNA electroporation could imply a need for repeated administration of the therapeutic cell product [61]. However, in the NK-92 model, permanent gene expression is not an inherent requirement, since NK-92 cells have a relatively short lifespan after administration [27]. In this regard, it is of critical importance to carefully consider the origin of the extracellular antigen-recognition domains. As repeated administration of CAR products containing murine-derived components can cause immunization and anaphylaxis, severely limiting safety and therapeutic efficacy [62], we have opted for the use of two fully-human CARs.

For clinical application, proliferation of NK-92 cells needs to be halted prior to infusion to avoid NK-92 cell engraftment in vivo, which could lead to the development of NK cell lymphoma. Although alternative methods are being examined [63], this “inactivation” step is most commonly performed by gamma-irradiation. We confirmed that 10 Gy gamma-irradiation effectively blocks NK-92 cell proliferation and leads to a gradual decrease in cell viability down to zero over the course of 1 week, paralleling the CAR expression kinetics after mRNA electroporation. Importantly, gamma-irradiation did not affect the functionality of the (dual) *CAR* mRNA-electroporated NK-92 cells, underlining the potential clinical applicability of the proposed therapeutic cell-based product.

Another potential advantage of using NK cells over the gold-standard CAR-T cells is the natural anti-tumoral activity of NK cells. However, this intrinsic cytotoxic capacity is largely dependent on exogenous activation stimuli, such as IL-2 [64]. IL-2 is an essential cytokine for NK cell growth and is, therefore, indispensable during NK-92 cell culture. IL-2 administration in humans can cause severe toxicity and can lead to regulatory T-cell activation, which is an undesired effect in the context of cancer immunotherapy due to their counterproductive inhibitory effect on cytotoxic lymphocytes [64]. Therefore, to pave the way towards clinical application, we omitted the supplementation of IL-2 during the last 24 h of NK-92 culture. As expected, this led to an almost complete abrogation of the natural cytotoxicity of NK-92 towards the NK-sensitive tumor cell line K562 [64–66]. In addition, as exemplified here both in the Daudi lymphoma cell line model as well as in the primary patient samples, some B-cell hematological malignancies are largely resistant to NK cell lysis [67]. Here, we show that CAR engineering of NK-92 cells overcomes the IL-2 dependence and restores their anti-tumor cytolytic activity. Corroborating the results of other dual-targeted CAR products [56, 68, 69], dual-CAR NK-92 were at least equally effective as their single-CAR counterparts in eliminating single and dual antigen expressing target cells, effectively reducing the probability of antigen escape. Interestingly, dual-CAR NK-92 display higher cytotoxicity towards CD19^−^BCMA^+^ cells compared to BCMA-CAR NK-92. The reason for this discrepancy, remains to be elucidated but the increased frequency of BCMA CAR^+^ cells in the dual-CAR NK-92 population compared to the BCMA-CAR NK-92 provides a likely explanation for the heightened lytic activity against BCMA^+^ target cells in the dual-CAR NK-92 conditions. Moreover, it was recently reported that dual CD19- and BCMA-CAR-T cells were able to completely ablate regulatory B-cells from the bone marrow of MM patients, contributing to a favorable environment for clearance of myeloma cells in the bone marrow [25]. Hence, in addition to their direct anti-tumor activity, our dual-CAR NK-92 cells could also play an important role in reshaping the tumor microenvironment.

The use of dual-CAR NK-92 cells as presented in this study contains some limitations. First, supplementating the NK-92 culture medium with animal serum instead of human serum limits the clinical translational potential. To the best of our knowledge, there have been no comparative studies between the two, leaving uncertainty on whether the serum source affects CAR NK-92 performance. Of interest, one study on serum-free NK-92 culture reports no significant difference in viability, proliferation, receptor expression levels, or perforin and granzyme levels, but a significantly decreased degranulation and cytotoxic potential in vitro which could be partly recovered after the addition of serum [70]. Second, our follow-up period of NK-92 cell viability and proliferation after irradiation was limited to 7 days. However, others have reported on the complete abrogation of NK-92 expansion for more than 30 days using the same irradiation protocol as described here [42, 71]. In our view, this provides sufficient proof for the safe clinical application of dual-CAR NK-92. Finally, we did not directly investigate potential “off-tumor” effects in our work. As discussed above, so far, NK-92 clinical studies have not revealed any toxicity towards normal cells or tissues. Moreover, given that “on-target/off-tumor” toxicities of current CD19- and BCMA-CAR-T products, such as hypogammaglobulinemia following B-cell depletion, are well described and manageable [1, 9], it seems unlikely that combinatorial targeting of these antigens will result in any unanticipated off-tumor side effects. Moreover, in the event such toxicities occur with our dual-CAR NK-92 approach, repeated administration of the cells can be terminated, benefiting from their limited persistence after irradiation and the transient nature of the CAR-encoding mRNA.

## Conclusions

In conclusion, we demonstrate the efficient generation of dual CD19- and BCMA-CAR expressing NK-92 cells. These dual-CAR NK-92 cells have potent and specific cytotoxic activity towards CD19^+^ and BCMA^+^ tumor cell lines, as well as primary B-ALL and MM cells. Gamma-irradiation was confirmed to be adequate to avoid proliferation and persistence of the NK-92 cells while maintaining functionality. Future work should be conducted to validate our findings in vivo before proceeding to early phase clinical testing to evaluate safety and feasibility. However, in the long term, we envision that our CD19/BCMA-targeted dual-CAR NK-92 can be used as an off-the-shelf therapeutic for B-cell leukemia, lymphoma and myeloma patients to address the current problems of antigen-negative and antigen-positive relapses after autologous single CAR-T administration.

## Supplementary Information

**Additional file 1.** Decomposition of CAR expression of single- and dual-CAR NK-92 cells. BCMA-CAR (left) and CD19-CAR (right) expression in each of the relevant NK-92 cells (N = 19)

**Additional file 2.** Dual-CAR expression kinetics after irradiation of dual-CAR NK-92. Follow-up of dual-CAR expression over the course of three days (N = 3).

## Data Availability

The datasets used and/or analyzed during the current study are available from the corresponding author on reasonable request.

## Abbreviations

ANOVA: Analysis of variance
BCMA: B-cell maturation antigen
BMMNC: Bone marrow mononuclear cells
CAR: Chimeric antigen receptor
CS: Co-stimulatory domain
ELISA: Enzyme-linked immunosorbent assay
E:T: Effector-to-target
GvHD: Graft-versus-host disease
H: Hinge domain
IL: Interleukin
IMDM: Iscove’s Modified Dulbecco’s Medium
IVT: In vitro transcription
MEM: Minimum essential medium
MM: Multiple myeloma
Rh: Recombinant human
scFv: Single-chain variable fragment
SD: Signaling domain
SP: Signal peptide
TM: Transmembrane domain
