# Supplementary figures and images for: Two for one: targeting BCMA and CD19 in B-cell malignancies with off-the-shelf dual-CAR NK-92 cells
Source: J Transl Med. 2022 Mar 14;20:124. doi: 10.1186/s12967-022-03326-6 (PMC8919645; doi:10.1186/s12967-022-03326-6)

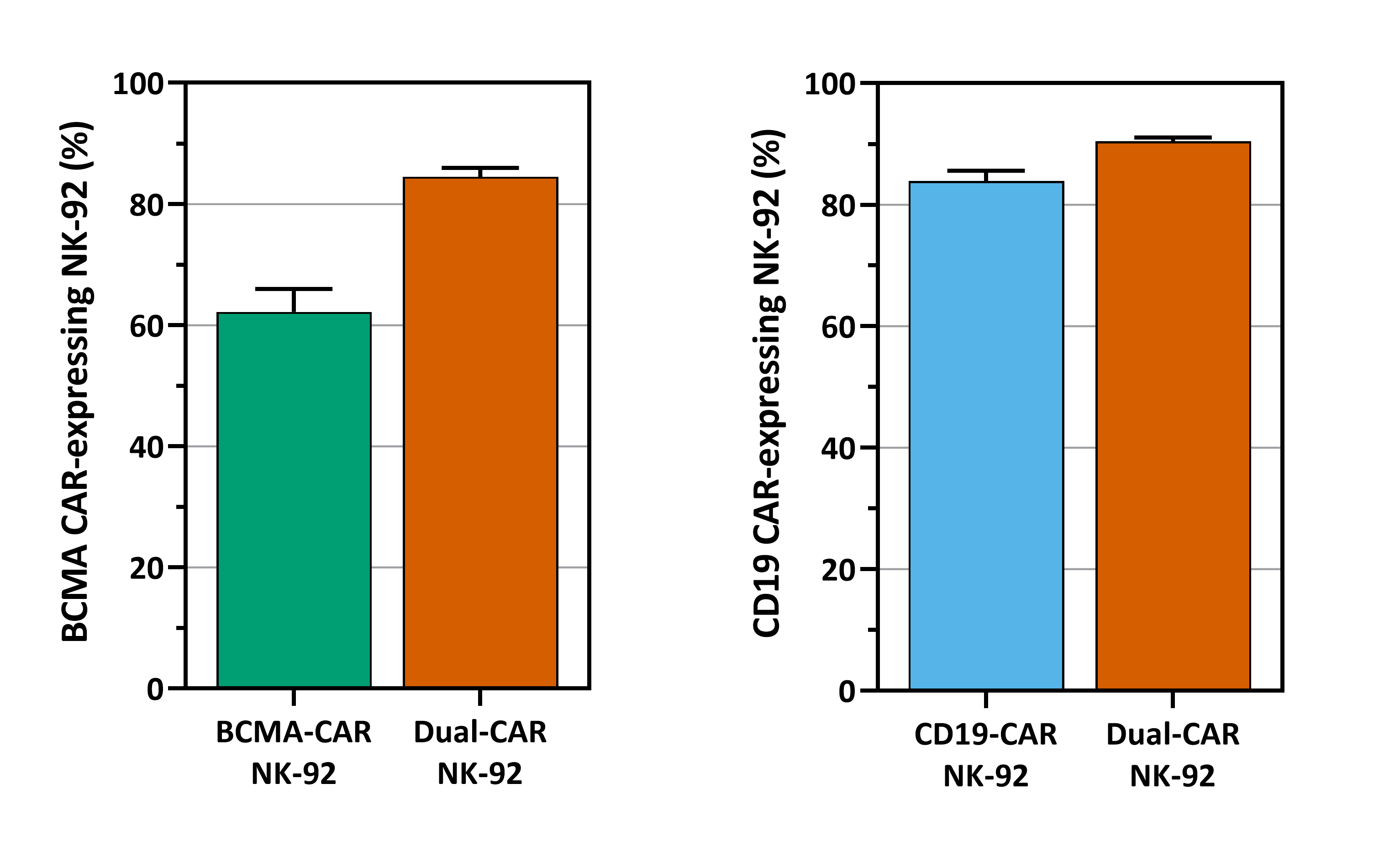

Supplement: Supplementary file 1 — Additional file 1. Decomposition of CAR expression of single- and dual-CAR NK-92 cells. BCMA-CAR (left) and CD19-CAR (right) expression in each of the relevant NK-92 cells (N = 19) [file 12967_2022_3326_MOESM1_ESM.tif]

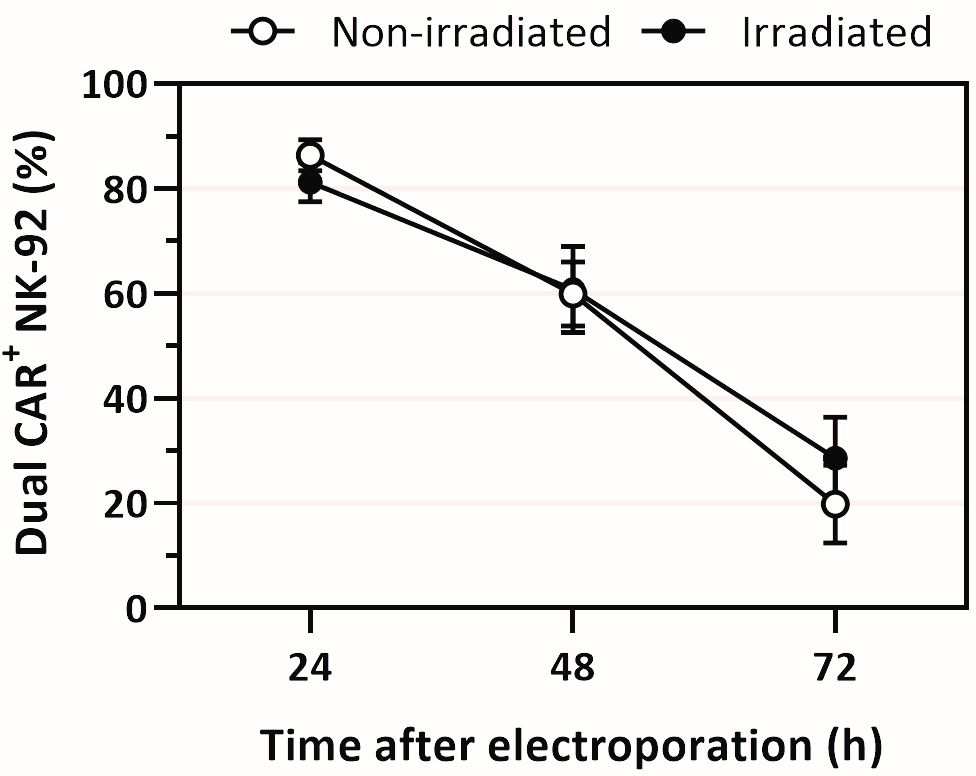

Supplement: Supplementary file 2 — Additional file 2. Dual-CAR expression kinetics after irradiation of dual-CAR NK-92. Follow-up of dual-CAR expression over the course of three days (N = 3). [file 12967_2022_3326_MOESM2_ESM.tif]
